# The Effect of Clinical Exercise Training on Plantar Pressure, the Subtalar Joint, and the Gait Cycle in Pregnant Women: Randomized Clinical Trial

**DOI:** 10.3390/jcm13247795

**Published:** 2024-12-20

**Authors:** Ayşe Kayalı Vatansever, Seçkin Şenışık, Dilek Bayraktar, Mehmet Demir, Fuat Akercan

**Affiliations:** 1 Department of Physiotherapy and Rehabilitation, İzmir Bakırcay University, 35665 Izmir, Turkey; ayse.vatansever@bakircay.edu.tr; 2 Sports Medicine Department, Ege University, 35040 Izmir, Turkey; seckinsnsk@gmail.com; 3 Orthopedics and Traumatology Department, Ege University Hospital, 35100 Izmir, Turkey; 4 Gynecology And Obstetrics Department, Ege University, 35040 Izmir, Turkey; mehmet.demir@ege.edu.tr (M.D.); fuat.akercan@ege.edu.tr (F.A.)

**Keywords:** women’s health, exercise, biomechanics, therapeutic exercise, core stability

## Abstract

**Background/Objectives**: This study aims to examine the effects of clinical exercise training on foot plantar pressure, the subtalar joint, and the gait cycle during pregnancy. **Methods**: The study was planned as a randomized, controlled, and single-blind study. Participants’ demographic information, obstetric evaluation, physical activity level, fall history, and pain evaluation were recorded. Foot plantar pressure, the subtalar joint, and the gait cycle were measured through pedobarography at Gait Laboratory. The control group was recommended walking. Clinical exercise training was given to the study group 2 days a week for eight weeks. Evaluations were made on day 0 and the day corresponding to the end of week 8. **Results**: The study was completed with 50 people in the study group (age: 29.7 ± 3.8 years) and 51 in the control group (age: 29.1 ± 6.1 years). As a result of the parametric and non-parametric tests performed before and after the exercise, it was observed that there was a statistically significant difference between the two groups in weight, BMI, pain score, static plantar pressure, dynamic plantar pressure, subtalar joint flexibility, duration of the walking period, and multistep walking speed (*p* < 0.01). The two groups had a significant difference only in the dominant midfoot plantar pressure (*p* > 0.05). **Conclusions**: According to our research, weight control and pain relief are observed in women who engage in clinical exercise in the second trimester of pregnancy; plantar pressure and subtalar joint flexibility are preserved, the walking period does not increase, and the multistep walking speed can be maintained after eight weeks.

## 1. Introduction

Changes in body biomechanics during pregnancy can lead to various musculoskeletal problems [1]. Common issues during this period include pain in the ankle, knee, and pelvic regions, as well as difficulties in walking [2]. Hormonal changes and fetal growth contribute to shifts in the center of gravity and pressure [3]. These changes have significant effects on plantar pressure, the gait cycle, and lower extremity joint mobility. Research indicates that these biomechanical changes begin as early as the first trimester of pregnancy [4]. As pregnancy progresses, changes, such as flattening of the foot structure, increased plantar pressure, and decreased ankle stability, become evident [5,6]. These alterations often lead to balance impairments and a higher risk of falls, especially during the later trimesters [3]. These changes can particularly challenge the maintenance of static and dynamic stability in the second and third trimesters. Furthermore, the duration of double-limb support increases, the walking speed decreases, and the stride length shortens as pregnancy advances [7]. Changes in the pelvic region also affect trunk movements and postural dynamics. Common postural adaptations include a backward tilt of the trunk and an anterior tilt of the pelvis in standing position [8]. Exercise has the potential to mitigate these changes by improving lower extremity strength, enhancing joint stability, and optimizing gait biomechanics [9]. As a result of these changes, the prevalence of pregnancy-related low back pain and/or pelvic girdle pain has been reported to be as high as 86% in the last trimester [10].

The role of regular exercise in preventing and managing these biomechanical problems during pregnancy is frequently investigated. Clinical exercise training is a physiotherapy-based program tailored to gestational age aiming to prevent mechanical pain and other pregnancy-related complaints [11,12]. Targeted exercise programs can enhance postural stability, reduce musculoskeletal strain, and improve functional outcomes during pregnancy. Evidence suggests that regular clinical exercise improves both physical and social quality of life and mitigates the adverse effects experienced during pregnancy [9]. This study aims to investigate the effects of clinical exercise programs on plantar pressure, the gait cycle, and subtalar mobility in healthy pregnant women. The research question is as follows: How do clinical exercise programs influence plantar pressure, the gait cycle, and subtalar joint flexibility during pregnancy, and what are the relationships among these changes? Our hypothesis is that regular clinical exercise programs improve plantar pressure and lower extremity mobility while reducing gait cycle imbalances and enhancing both static and dynamic stability.

## 2. Materials and Methods

### 2.1. Design

**Figure 1:**
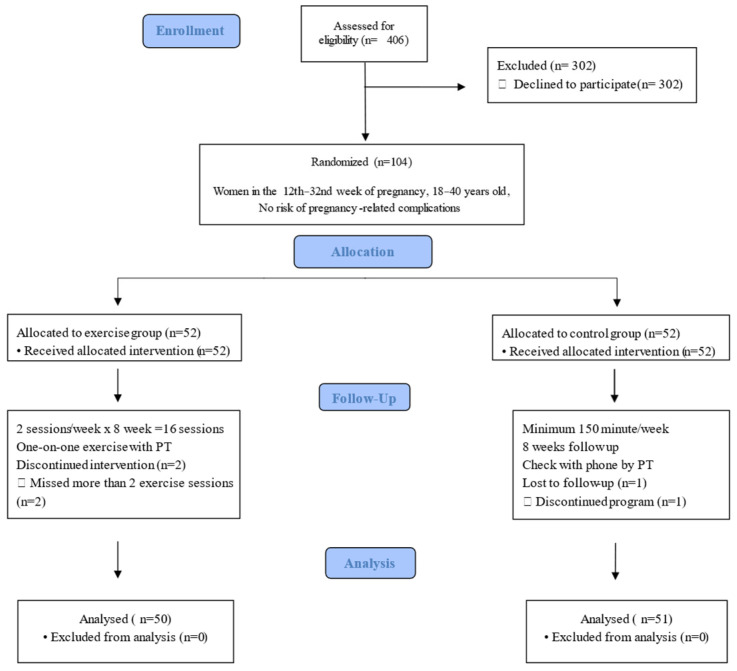
Flow chart of study.

This single-blind, randomized controlled clinical trial [13] evaluated the effectiveness of a clinical exercise program on static and dynamic plantar pressure, subtalar joint flexibility, walking speed, and walking duration in pregnant women. Participants were blinded to group allocation, while investigators were aware of the groups (Figure 1). The study was conducted following the CONSORT guidelines.

### 2.2. Participants

Healthy pregnant women who applied to the Gynecology and Obstetrics Outpatient Clinic of Ege University Medical Faculty Hospital participated in the study. The research was conducted between 25 April 2022 and 15 May 2023. Inclusion criteria were age between 18 and 40 years, no risk of pregnancy-related complications, and pregnancy between the 12th and 32nd week of gestation, while exclusion criteria were a history of lower extremity, pelvic, or spine surgery and/or pain for more than six months and any fetal developmental delay. The participants were informed about the study, and written consent forms were obtained from them at the Ege University Sports Medicine Department.

### 2.3. Intervention

The control group underwent at least 150 min of moderate-intensity aerobic exercise program as specified in the American College of Obstetricians and Gynecologists (ACOG) guidelines. They walked for 30 min a day, five days a week, according to their physical activity level. Exercise intensity was adjusted by 2–3 points according to the rate of perceived exertion (RPE) using the Modified Borg Scale [14]. Participants were called by phone once a week to check whether they were exercising.

For the exercise group, a one-on-one clinical exercise program was applied with a physiotherapist for a total of 16 sessions two days a week for eight weeks. Each session lasted 45 min. A clinical exercise program suitable for pregnant women was organized by paying attention to the week of pregnancy [2,15,16]. The clinical exercise program included core stabilization exercises, exercises involving the pelvic floor muscles and the muscles surrounding the hip, and therapeutic exercises combined with breathing to strengthen the lower extremity muscles (Supplementary Materials). Each session included warm-up–load–cool-down periods. Free weights, resistance bands, or body weights were preferred for strengthening exercises [15]. According to the recommendations of the American College of Sports Medicine (ACSM), strength training with an elastic band was adjusted according to the RPE. According to the Modified Borg Scale, loading exercises were performed with a score of 2–3, corresponding to moderate-intensity exercise [14].

### 2.4. Assessments

For the demographic assessment, the age, educational status, dominant side, height, body weight, and body mass index (BMI) of the pregnant women were included. They were asked to kick a soccer ball to determine their dominant side [17]. The preferred limb for kicking the ball was recorded as dominant. Height was measured using a stadiometer in a standing position without shoes. Body weight (BW) was measured without shoes and with a clinical measuring device. All of these measurements were performed by a single person. BMI was calculated as body weight in kilograms divided by square height in meters. The unit was recorded as kg/m^2^. For the obstetric assessment, the gestational week and the number of pregnancies were recorded. Physical activity levels of pregnant women were measured using the International Physical Activity Questionnaire—Short Form (IPAQ-SF). The questionnaire asks about walking, moderate-intensity and vigorous-intensity exercises, and sitting time in minutes and frequency in the last week. The physical activity score was obtained by calculating the person’s answers to these seven questions in MET-min/w (metabolic equivalent—minutes/week) [18].

### 2.5. Outcome Measurements

For the pedobarography assessment, measurements were performed with the Materialise Motion Footscan^®^ v9 Scientific (Rsscan International, Paal, Belgium, 40 × 100 cm, 300 Hz) Pedobarography measurement system. Participants were first walked on the test track for familiarization. The device automatically calculated the average data by taking five records for each foot [19]. The static measurement was recorded while the volunteer was in an upright and immobile position with both feet touching the platform and feet shoulder-width apart. The subject was asked to balance on both feet for three repetitions of thirty seconds each. Each measurement was recorded, and the average of the three measurements was taken. The participant rested for one minute between measurements. The plantar foot loading pressures were recorded as a percentage. Subtalar joint flexibility (degrees) was measured. The foot full period (ms), plantar pressure areas (%), and multistep walking speed (km/h) were measured during the gait cycle. The measurements were performed by the same researcher.

### 2.6. Other Measurements

For the pain assessment, the presence, location, and intensity of pain were questioned. The severity of pain was evaluated with the Visual Analog Scale (VAS). A straight line was drawn on a piece of paper, and 0 was marked as “no pain” and 10 as “unbearable severe pain”. The participant was asked to mark on the paper, and then the mark was measured with a ruler, and the corresponding number was considered the pain intensity score.

### 2.7. Sample Size, Randomization, and Blinding

In order to find a significant difference between the gait analysis results obtained before and after exercise in the exercise and control groups, a power analysis was performed using Gpower 3.1.2 under a repeated measurement variance analysis test. In the analysis, α = 0.05 and a medium effect size f = 0.25 were taken with 80% power, and a total of 98 individuals, 49 in each group, were found sufficient. Considering the loss of data in the follow-up, it was decided to recruit 5% more patients. Thus, 104 (52:52) was determined as the final sample size.

The randomization algorithm (Maximum Allowed % Deviation = 10%) was applied using PASS software 11.0 (NCSS LLC, Kaysville, UT, USA) to generate a randomization list that would allow participants to be assigned to two groups of 52 each.

After obtaining the consent form, the demographic information, obstetric information, physical activity levels, and presence, score, and location of pain were evaluated. Afterward, a gait assessment was performed with a pedobarography device in the Gait Analysis Laboratory of Ege University Faculty of Medicine, Department of Orthopedics and Traumatology. After randomization, the participants were divided into groups. The study was planned as a single-blind, randomized controlled trial. The participants continued the study without knowing which group they were in. All assessments were performed twice using the same method on day 0 and at the end of the following eight weeks. It was performed between 09:00 and 12:00 with comfortable clothing to avoid being affected by fatigue. Participants in the exercise group were excluded from the study when they could not attend two classes in a row. Participants in the control group were excluded from the study when they did not complete the walking program for a total of at least 150 min in the last week.

### 2.8. Statistical Analysis

Numerical data were summarized using mean, standard deviation, median, minimum, and maximum values, and categorical data were summarized using frequency and ratio values with the IBM SPSS Statistics 25.0 (IBM SPSS Statistics for Windows, Armonk, NY, USA: IBM Corp.) package program. The normality of numerical data distribution was assessed using the Shapiro–Wilk test, which confirmed that the data followed a normal distribution. Numerical demographic data were compared between the study groups using an independent sample *t*-test or Mann–Whitney U; categorical data were compared using the Pearson Chi-square test or Fisher’s exact probability test.

Parametric and non-parametric models with group x time interaction were used to test whether the temporal change in pedobarographic measurements varied between groups. In these analyses, some of the pain, subtalar joint flexibility, and gait period measurements were analyzed through the non-parametric approach of the Brunner–Langer model (F1-LD-F1 design). Other measurements were analyzed with linear mixed models (LMMs) with a parametric approach (this LMM used a random cut-off point, an unstructured covariance matrix, and heterogeneous residual variance for groups). Subgroup analyses for interactions were performed within the same analysis approach. A value of *p* < 0.05 was considered statistically significant. All statistical analyses were performed using R software (R software, version 4.0.5, package: arsenal; R Foundation for Statistical Computing, Vienna, Austria) and SAS software (version 9.3; packages: PROC MIXED; SAS Institute, Cary, NC, USA).

## 3. Results

This clinical trial was completed with the participation of 101 volunteer pregnant women. The descriptive and clinical characteristics of the participants are presented in Table 1. Two participants in the study group did not want to attend two sessions or more in a row. Therefore, the exercise group was completed with 50 participants. One woman in the control group did not want to do the walking program for a week and left the study. The control group was completed with 51 participants.

### 3.1. Primary Outcomes

An interaction between group and time was observed in the analysis investigating whether the BW change was similar in the groups (interaction *p* < 0.0001). Then, the groups were compared at baseline and in change differences (week 8 BW—week 0 BW). While there was no difference between the groups at baseline (*p* = 0.584), there was a difference in body weight in both groups at the end of the study (*p* < 0.0001 for both groups). The mean weight gain was 3.9 kg in the exercise group and 5.7 kg in the control group (*p* < 0.0001) (Table 1).

In all parameters, there was no statistical difference between the two groups at baseline (*p* > 0.05). Group–time interaction was significant in the change analysis of these parameters (interaction *p* < 0.001). At the end of the eighth week, it was observed that the dominant static plantar pressure increased more in the control group than in the exercise group, while the non-dominant static plantar pressure decreased more (*p* < 0.001). At the end of the eighth week, the difference between the dominant forefoot plantar pressure values of the two groups was statistically significant (*p* < 0.001). Although non-dominant forefoot plantar pressure increased in both groups, the increase was greater in the control group. This change was statistically significant between the two groups (*p* < 0.001). When midfoot plantar pressure was examined, it was found that there was an increase in both groups in the non-dominant foot, and this increase was significant only in the control group (*p* < 0.001). On the dominant side, there was a decrease in the exercise group and an increase in the control group. At the end of the study, hindfoot plantar pressure was statistically significantly higher in the exercise group than in the control group on both the dominant and non-dominant sides (*p* < 0.0001) (Table 2).

Dominant subtalar joint flexibility showed no change in the exercise group and a decrease in the control group (interaction *p* < 0.0001). Non-dominant subtalar joint flexibility increased slightly in the exercise group but decreased in the control group. Dominant foot gait period duration increased significantly in both the exercise group and the control group at the end of the study (*p* < 0.0001), and this increase was statistically significantly greater in the control group (*p* < 0.001). Non-dominant gait period duration increased statistically significantly in both groups (*p* < 0.0001). The rate of increase in the control group was higher than in the exercise group. Multistep walking speed showed a statistically insignificant increase in the exercise group at the end of eight weeks (*p* = 0.085), while a significant decrease was found in the control group (*p* < 0.0001) (Table 3).

### 3.2. Secondary Outcomes

**Figure 2:**
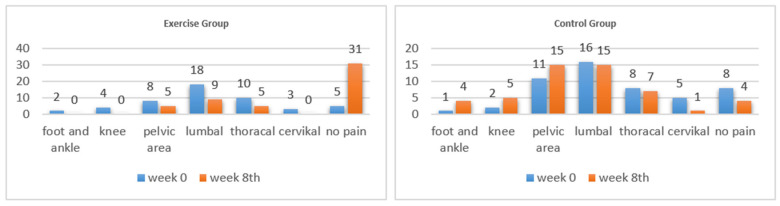
Pain scores and localization in exercise and control groups.

Pain scores increased statistically significantly in the control group and decreased in the exercise group (interaction *p* < 0.0001). While the pain level of the exercise group was higher than that of the control group at baseline (*p* = 0.023), the pain level of the control group was higher than that of the exercise group at week 8 (*p* < 0.001) (Table 4). In the exercise group, while 5 people had no pain at the beginning, 31 had no pain in the eighth week. The lumbar region in both groups was the most common site of pain localization. The second most common pain localization site was in the thoracic and pelvic regions (Figure 2).

## 4. Discussion

In this study, which examined the effects of a clinical exercise program during pregnancy on plantar pressure, subtalar joint flexibility, and gait cycle, we found that static and dynamic plantar pressures were preserved. Subtalar joint flexibility did not decrease despite the progression of pregnancy, walking speed was maintained, and walking time remained unchanged. Furthermore, the study demonstrated that pain levels in pregnant participants improved, with a significant reduction in pain scores.

Clinical exercise aimed at maintaining and improving general health often includes physical rehabilitation [20]. However, the literature on physical activity and exercise during pregnancy remains limited [21]. In our study, we found that participants in a clinical exercise program during pregnancy experienced less weight gain and had lower BMI values compared to those who did not engage in such programs.

The percentages of static plantar pressure during standing are typically expected to be equally distributed between the dominant and non-dominant foot [22]. At the end of the 8-week study, the control group showed an 8.2% increase in static plantar pressure on the dominant side, accompanied by an equivalent decrease on the non-dominant side. In contrast, the exercise group exhibited only a 1% change in static plantar pressure. The dominant foot, often regarded as having superior neuromuscular function, is thought to adapt to the changing balance strategies during pregnancy by increasing static loading on the dominant side to maintain stability [23,24]. We believe that the smaller increase in static plantar pressure observed in the exercise group was due to the stabilizing effects of clinical exercise. Static plantar pressure measurement, widely recognized as a determinant of postural stability, is among the most commonly used clinical assessments. To stabilize the body against gravity and maintain balance during various motor tasks, the central nervous system utilizes biomechanical strategies and neuromuscular adaptations. During standing, it adjusts postural parameters in the sagittal plane, optimizing energy consumption through feedback transmitted via plantar pressure [25]. According to a review by Conder et al., instability during pregnancy, particularly in the second trimester, is attributed to weak somatosensory inputs rather than anatomical changes [26]. Considering these findings, it can be inferred that the clinical exercises performed by the pregnant women in the exercise group minimized static plantar pressure by enhancing postural stability. Monteiro et al. [27] reported that regular exercise strengthens the intrinsic foot muscles, reducing plantar pressure and supporting the foot arch. These effects likely contributed to the observed improvements in postural stability within the exercise group. This observation suggests that walking alone may be insufficient to maintain stability during pregnancy.

Dynamic plantar pressures refer to the pressures exerted on the plantar surface of the foot during the gait cycle [28]. With the rapid increase in BMI during pregnancy, differences in plantar pressure distribution between both feet are expected. As the abdominal region expands forward, the center of gravity (COM) shifts accordingly [29]. In our study, the control group, which did not engage in exercise, exhibited a statistically significant increase in forefoot plantar pressure and a decrease in hindfoot plantar pressure. In contrast, the exercise group demonstrated an increase in forefoot plantar pressure on the dominant side and a decrease in hindfoot plantar pressure, with no significant change observed in midfoot plantar pressure. Previous studies investigating plantar pressure changes during pregnancy have reported varying results. Consistent with our findings, one study observed an increase in forefoot plantar pressure in both extremities [30]. Similarly, Lee et al. reported a decrease in lower extremity and plantar flexor muscle strength accompanied by an increase in plantar pressure [31]. Conversely, Maslon et al. identified a positive correlation between weight gain during pregnancy and midfoot plantar pressure [32]. Unver et al. [33] noted that foot exercises improved plantar pressure distribution by strengthening the muscles supporting the foot arch. This aligns with our findings, where the exercise group demonstrated improved plantar pressure management compared to the control group. Contrary to these findings, other studies have suggested that midfoot plantar pressure increases as the medial longitudinal arch lowers with the progression of gestational weeks [34]. Ribeiro et al., in their review, reported that the maximum plantar force on the hindfoot increased by 15% on the right foot and 10% on the left foot [35]. These discrepancies highlight how pressure changes in one area of the plantar surface can differentially affect other regions. The variation in results between studies, as well as the differences in plantar pressure distributions between exercisers and non-exercisers, may be attributed to these complex interrelationships. In our study, the observed increase in pressure in the forefoot and midfoot regions may have contributed to a corresponding decrease in hindfoot plantar pressure. Notably, one study suggested that optimal pressure distribution for maintaining foot stability during walking requires higher hindfoot pressure relative to midfoot pressure [36]. Buldt et al. [36] emphasized that foot structure significantly influences plantar pressure distribution and highlighted the role of targeted exercises in optimizing this distribution. In this context, the exercise program implemented in this study may have played a role in increasing hindfoot plantar pressure, thus supporting stability in pregnant women.

The subtalar joint is challenging to evaluate due to its complex interaction with the ankle and transverse tarsal joints and its direct association with foot and ankle deformities [37]. At the end of the study, we observed a decrease in subtalar joint flexibility in the control group, while no significant change was noted in the exercise group. Forczek et al. reported a five-degree reduction in ankle dorsiflexion during walking in pregnancy [34]. Similarly, another study found decreased ankle plantarflexion and dorsiflexion in the second and third trimesters of pregnancy [36]. Such reductions are likely to contribute to a decrease in subtalar joint flexibility. Clinical exercise during pregnancy has been shown to help manage body weight and improve soft tissue flexibility [38]. Regular exercise can support the maintenance of joint flexibility throughout the lower extremities, including the subtalar joint. In our study, clinical exercise programs appear to have preserved subtalar joint flexibility by targeting the muscles that stabilize the ankle and the foot. While walking provides natural subtalar joint movements, such as pronation and supination, the exercise program’s broader approach to strengthening the surrounding musculature likely prevented the flexibility losses observed in the control group [34]. This suggests that clinical exercise programs may play a key role in preserving subtalar joint flexibility during pregnancy, thereby mitigating potential biomechanical limitations.

Walking is recognized as one of the most challenging functions during pregnancy [39]. In this trial, the duration of the walking period increased in both groups and on both sides, with a greater increase observed in the control group. A study by Sadeghi et al. found that stance time was longer on the non-dominant side [40]. Similarly, in this study, the non-dominant gait period duration was longer in the non-exercising group. Pregnant women are reported to extend the gait period duration as a strategy to maintain stability during walking [4]. This is often achieved by increasing the duration of the double support phase and decreasing the duration of the single support phase, with a shorter rocking phase and a prolonged stance phase [5]. In contrast, the clinical exercise group demonstrated a smaller increase in gait period duration, likely due to enhanced subtalar joint stability and improved neuromuscular control resulting from the exercise program [41]. The smaller increase in gait period duration observed in the exercise group compared to the control group may be attributed to improved static and dynamic stability as a result of the clinical exercise program. Additionally, we found that walking speed decreased in the control group but was maintained in the exercise group. Stronger foot and ankle muscles, developed through the exercise program, may have supported efficient load distribution and reduced medio-lateral instability during walking [33]. According to Maslon et al., the medio-lateral component of ground reaction forces is compensated by the foot during walking, leading to a lower walking frequency and reduced stride length in pregnant women [32]. These findings highlight the potential of clinical exercise programs to counteract pregnancy-related biomechanical changes, thus promoting a more stable and efficient gait pattern.

The localization and onset of pain during pregnancy can vary among individuals. Musculoskeletal pain is generally expected to begin around the 12th week of gestation due to hormonal changes and is most commonly reported in the lumbar and pelvic regions [40]. At the beginning of this study, the lumbar and pelvic regions were the primary sites of pain complaints in pregnant women, with 36% of participants in the exercise group and 31% in the control group reporting low back pain. Similarly, pain complaints were observed in 22% of the control group and 16% of the exercise group. Ostgaard et al., in a randomized controlled trial involving 362 healthy pregnant women, demonstrated that antenatal education and exercise could prevent musculoskeletal pain during pregnancy, reduce pain intensity in those already experiencing discomfort, and avoid the onset of new pain [42]. By the end of the study, pain in various regions, including the lower extremities, increased in the control group, whereas pain complaints in areas like the foot, ankle, knee, and neck resolved in the exercise group. Additionally, the mean pain score decreased across the exercise group. These findings highlight the role of regular clinical exercise in reducing pregnancy-related musculoskeletal pain and suggest that such programs can contribute to improving overall physical well-being during pregnancy by addressing pain and enhancing mobility.

This study evaluates the role of clinical exercise programs in managing biomechanical changes during pregnancy. Improvements in subtalar joint flexibility, balanced plantar pressure distribution, and enhanced gait mechanics significantly contribute to reducing common musculoskeletal issues during pregnancy. However, this study has certain limitations. For instance, edema, which can influence plantar pressure distribution, was not assessed in this study. Pregnancy-related edema may alter plantar pressure and lead to potential inaccuracies in biomechanical analyses [43]. The absence of detailed evaluations of biomechanical factors, such as lumbopelvic angulation, which can impact plantar pressure and the gait cycle, also represents a limitation [44]. Additionally, pre-pregnancy exercise habits were not evaluated, which may have influenced participants’ adaptation to the program and their recovery rates. While measurements were conducted objectively using a gait analysis system, perceived exertion levels and pain scores were assessed subjectively. This subjective assessment could have been influenced by participants’ perceptions, potentially leading to underreporting of fatigue or pain levels. To address this, heart rate and oxygen saturation were monitored via pulse oximeter during exercise sessions, providing additional objective data. Although the sample size is sufficient for a randomized, controlled, single-blind design, studies involving larger populations and longer intervention periods could enhance the generalizability of the findings. Future research should consider evaluating long-term effects on biomechanical adaptations across different stages of pregnancy. Moreover, objective assessments of muscle strength, flexibility, edema, and lumbopelvic angulation could provide more comprehensive insights. Such studies could also investigate the effects of clinical exercise programs on musculoskeletal health in the postpartum period, exploring their potential role in supporting recovery processes after childbirth. These findings and recommendations could serve as a foundation for expanding prenatal and postnatal care protocols.

## 5. Conclusions

This study investigated the effects of a clinical exercise program during pregnancy on plantar pressure, subtalar joint flexibility, and walking speed. Regular participation in the exercise program minimized changes in these parameters and contributed to better outcomes compared to non-exercising pregnant women. Reduced weight gain, alleviation of pain complaints, and lower pain levels in the later weeks of pregnancy were observed, supporting the healthy progression of pregnancy. These findings highlight the potential of clinical exercise programs to preserve muscle and joint flexibility, enhance muscle strength, and maintain walking speed during pregnancy. Multidimensional programs incorporating core stabilization, upper and lower extremity exercises, and breathing techniques can enhance neuromuscular control, improve joint stability, and maintain functional mobility. Supervised by physiotherapists and obstetricians, such programs can include balance training, strengthening, and stretching routines tailored to the physical condition, gestational stage, and activity level of each individual. By proactively addressing biomechanical changes, clinical exercise programs can help pregnant women maintain mobility, facilitate the birthing process, and reduce the risk of musculoskeletal complications. However, while these results provide valuable insights into the benefits of exercise in mitigating pregnancy-related musculoskeletal changes, further studies are needed to evaluate the influence of factors like biomechanical structure, muscle strength, and edema on plantar pressures, joint flexibility, and pain levels during pregnancy.

## Figures and Tables

**Table 1:** Descriptive characteristics of the participants.

	Exercise Group (n = 50) M ± SD	Control Group (n = 51) M ± SD	*p* Value
Age (years)	29.7 ± 3.8	29.1± 6.1	0.505 ^1^
Height (cm)	164.0 ± 6.8	161.7± 6.7	0.084 ^1^
BW (kg)	66.0 ± 8.4	65.0± 9.8	0.584 ^1^
BMI (kg/m^2^)	24.5 ± 2.2	24.9 ± 3.4	0.491 ^1^
IPAQ-SF (MET-min/w)	Median (min–max) 290.8 (250.5–331.1)	Median (min–max) 279.5 (155.8–385.2)	0.061 ^4^
Gestation week	15.7 ± 3.1	14.8 ± 2.6	0.166 ^4^
Dominant Leg	n (%)	n (%)	0.063 ^2^
Right	49 (98%)	48 (96%)	
Left	1 (2%)	2 (4%)	
Number of children	n (%)	n (%)	0.273 ^3^
0	35 (70%)	29 (56.9%)	
1	12 (24%)	14 (27.5%)	
2	3 (6%)	5 (9.8%)	
3	0	3 (5.9%)	

M: mean; SD: standard deviation; cm: centimeter; n: the number of participants; kg: kilogram, BW; body weight; BMI: body mass index; IPAQ-SF: International Physical Activity Questionnaire—Short Form; MET: metabolic equivalent; min: minute; w: week. ^1^ Independent sample *t*-test, ^2^ Pearson Chi-square test, ^3^ Fisher’s exact probability test, ^4^ Mann–Whitney U test.

**Table 2:** Comparison of foot plantar pressures by group and time.

Plantar Pressure Measures	Dominance	Exercise Group (Baseline)	Exercise Group (Week 8)	Control Group (Baseline)	Control Group (Week 8)	*p*-Value (Group)	*p*-Value (Time)	*p*-Value (Interaction)
Static	ND	48.4 ± 5.2	47.4 ± 4.0	48.4 ± 6.5	40.2 ± 7.1	0.0017	<0.0001	<0.0001
	D	51.6 ± 5.2	52.6 ± 4.0	51.6 ± 6.5	59.8 ± 7.1	0.0017	<0.0001	<0.0001
Forefoot	ND	55.3 ± 2.2	56.5 ± 3.8	55.3 ± 3.5	58.4 ± 2.7	0.1148	<0.0001	<0.0001
	D	55.7 ± 2.6	55.3 ± 3.1	56.3 ± 3.3	59.4 ± 3.5	0.0002	<0.0001	<0.0001
Midfoot	ND	17.4 ± 4.8	17.6 ± 5.0	18.3 ± 4.7	20.3 ± 2.2	0.0211	0.0015	0.0078
	D	18.5 ± 5.0	17.9 ± 4.9	17.7 ± 4.6	19.0 ± 2.2	0.8801	0.2034	0.0010
Hindfoot	ND	27.3 ± 3.2	25.9 ± 1.9	26.4 ± 2.3	21.3 ± 1.6	<0.0001	<0.0001	<0.0001
	D	25.7 ± 2.6	26.8 ± 3.0	26.0 ± 2.3	21.6 ± 2.3	<0.0001	<0.0001	<0.0001

M: mean; SD: standard deviation; D: dominant; ND: non-dominant.

**Table 3:** Comparison of lower extremity kinematics and subtalar joint flexibility.

Measure	Dominance	Exercise Group (Baseline)	Exercise Group (Week 8)	Control Gorup (Baseline)	Control Group (Week 8)	*p*-Value (Group)	*p*-Value (Time)	*p*-Value (Interaction)
Subtalar joint flexibility	ND	12.5 ± 5.9	13.5 ± 5.3	12.1 ± 7.0	7.7 ± 4.6	<0.001	0.005	<0.0001
	D	12.7 ± 6.3	12.4 ± 4.0	12.9 ± 7.5	7.2 ± 5.5	<0.001	<0.0001	<0.0001
Walking period	ND	754.3 ± 62.2	772.6 ± 70.2	764.5 ± 99.8	838.3 ± 98.5	0.015	<0.0001	<0.0001
	D	743.5 ± 70.3	773.1 ± 85.5	749.5 ± 86.4	812.1 ± 81.8	0.050	<0.0001	<0.001
Multistep walking speed		3544.6 ± 367.2	3662.1 ± 363.8	3558.8 ± 484.9	2897.9 ± 638.3	<0.0001	<0.0001	<0.001

M: mean; SD: standard deviation; D: dominant; ND: non-dominant.

**Table 4:** Analysis of pain score values between groups.

	Exercise Group (n = 50) M ± SD	Control Group (n = 51) M ± SD	*p* Value
VAS—week 0	4.7 ± 1.8	3.9 ± 1.7	0.023 ^1^
VAS—week 8	2.2 ± 2.2	5.8 ± 1.9	<0.001 ^1^

^1^ Mann–Whitney U Test; M: mean; SD: standard deviation.

## Data Availability

The data presented in this study are available upon request from the corresponding author due to the principles of the Personal Data Protection Law in Turkey.

## Supplementary Materials

The following supporting information can be downloaded at https://www.mdpi.com/article/10.3390/jcm13247795/s1. Figure S1: Exercise training examples with a large exercise ball (A: shoulder mobility exercise; B: shoulder elevation exercise; C: scapular abduction exercise; D: Pelvic Floor Exercises); Figure S2: upper extremity strengthening training examples with a large exercise ball (A: shoulder flexion strengthening; B: shoulder adduction exercise in flexion; C: serratus anterior strengthening + thoracic–cervical mobility); Figure S3: Standing free weight exercise training examples (A: Heel raise + with elbow flexion; B: mini squat + shoulder flexion; C and D: deep squat exercise + overhead press). Figure S4: stretching exercise training examples on the mat (A: lateral trunk flexion; B: lower extremity butterfly position; C: upper extremity, spine, and pelvic circumference stretches; D: pelvic and lower extremity circumference stretching exercises).
