# Supplementary material for: The Effect of Clinical Exercise Training on Plantar Pressure, the Subtalar Joint, and the Gait Cycle in Pregnant Women: Randomized Clinical Trial
Source: J Clin Med. 2024 Dec 20;13(24):7795. doi: 10.3390/jcm13247795 (PMC11728308; doi:10.3390/jcm13247795)
Supplement: Supplementary file 1 [file jcm-13-07795-s001.zip › jcm-3358306-supplementary.pdf]

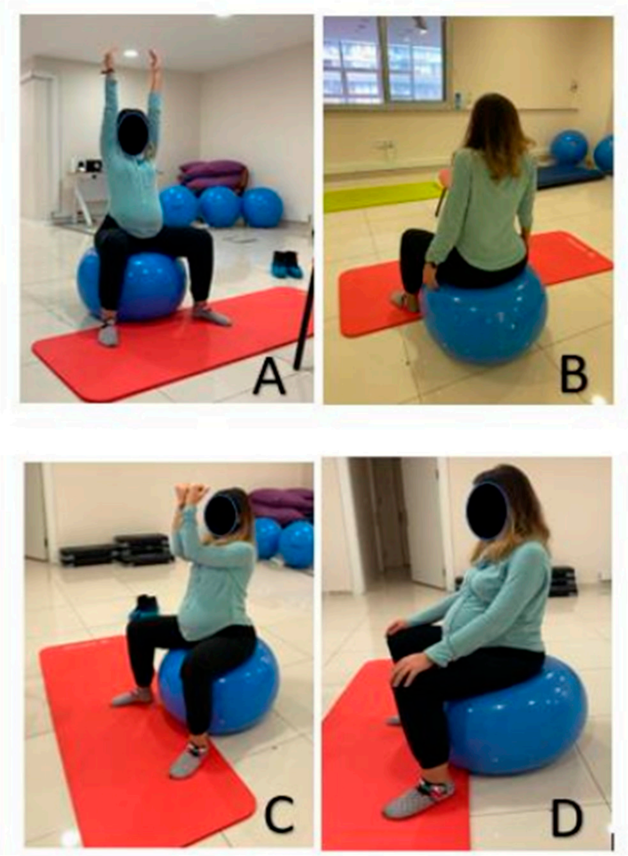


Figure S1: Exercise training examples with a large exercise ball (A: shoulder mobility exercise; B: shoulder elevation exercise; C: scapular abduction exercise; D: Pelvic Floor Exercises

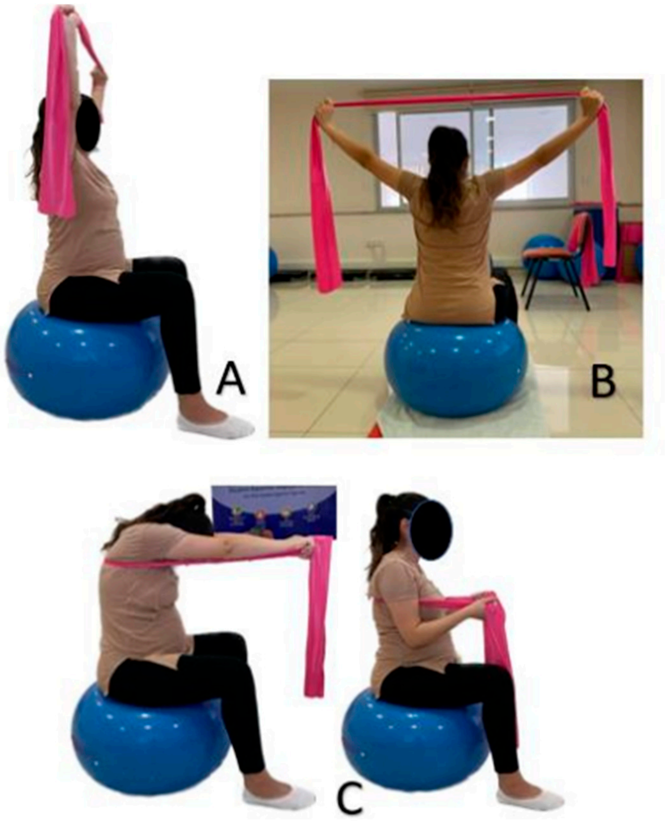


Figure S2: upper extremity strengthening training examples with a large exercise ball (A: shoulder flexion strengthening; B: shoulder adduction exercise in flexion; C: serratus anterior strengthening + thoracic–cervical mobility)

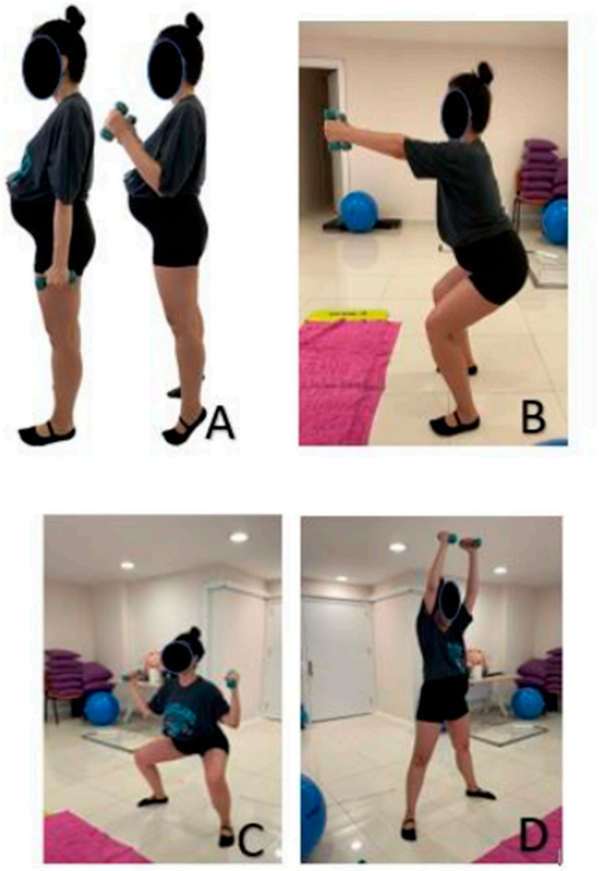


Figure S3: Standing free weight exercise training examples (A: Heel raise + with elbow flexion; B: mini squat + shoulder flexion; C and D: deep squat exercise + overhead press)

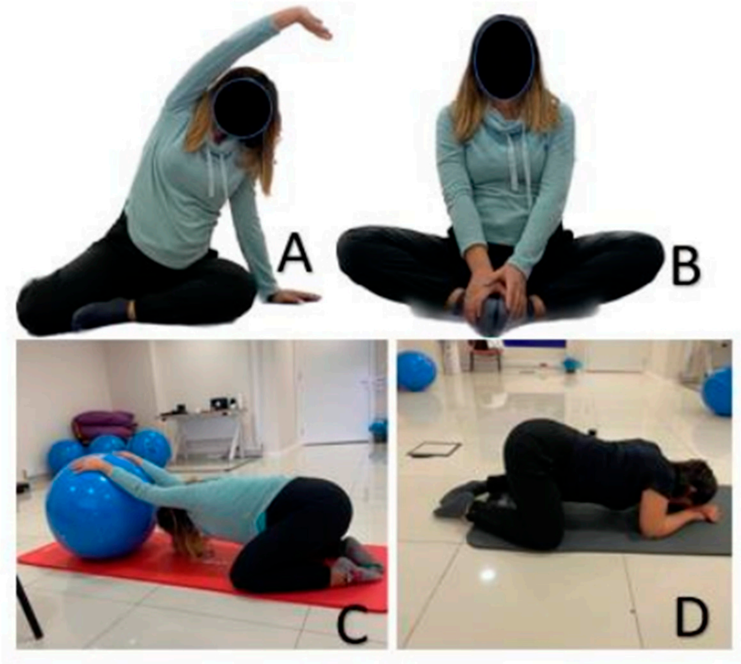


Figure S4: stretching exercise training examples on the mat (A: lateral trunk flexion; B: lower extremity butterfly position; C: upper extremity, spine, and pelvic circumference stretches; D: pelvic and lower extremity circumference stretching exercises).
